# Permutation Entropy and Its Niche in Hydrology: A Review

**DOI:** 10.3390/e27060598

**Published:** 2025-06-03

**Authors:** Dragutin T. Mihailović

**Affiliations:** Department of Physics, Faculty of Sciences, University of Novi Sad Dositej Obradovic Sq. 3, 21000 Novi Sad, Serbia; guto@df.uns.ac.rs

**Keywords:** complex systems, complexity, permutation entropy, hydrology, time series, AI based models in hydrology

## Abstract

One effective method for analyzing complexity involves applying information measures to time series derived from observational data. Permutation entropy (PE) is one such measure designed to quantify the degree of disorder or complexity within a time series by examining the order relations among its values. PE is distinguished by its simplicity, robustness, and exceptionally low computational cost, making it a benchmark tool for complexity analysis. This text reviews the advantages and limitations of PE while exploring its diverse applications in hydrology from 2002 to 2025. Specifically, it categorizes the uses of PE across various subfields, including runoff prediction, streamflow analysis, water level forecasting, assessment of hydrological changes, and evaluating the impact of infrastructure on hydrological systems. By leveraging PE’s ability to capture the intricate dynamics of hydrological processes, researchers can enhance predictive models and improve our understanding of water-related phenomena.

## 1. Introduction

### 1.1. What Is Complex System?

**Figure 1:**
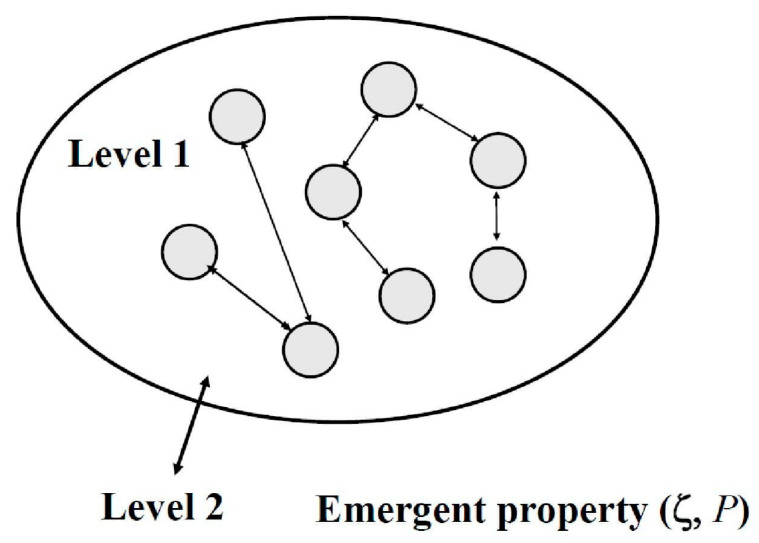
Sketch of an emergent property. At level I interactions occur. When describing the entity at level II, a new property appears. This property must have an associated set of data ζ and the probability distribution P that produce it. (Reproduced from [4]).

When we ask a question about the definition of a complex system, rhetorically or not, we are faced with whether the existing language of science can describe a world of complex systems in which there is a rule—“more is different” [1]. We consider a complex system (Figure 1) as it was done in Mihailović et al. [2], i.e.,: “… as a collection of entities (circles). Each component interacts with others via simple local rules and the possibility of feedback (arrows). When they interact, a new feature arises (level 1). The complex system cannot be decomposed nontrivially into a set of basic entities for which it is the logical sum [3] (to have the character of an emergent phenomenon, this new feature is completely unexpected [level 2]). The new property is characterized by (ζ, P), where ζ represents data, and P is the probability distribution”. It should be noted that P denotes a general probability distribution, not a single specific or concrete probability. This is perhaps the most concise definition that describes a complex system’s nature but does not indicate how to interpret that system holistically, considering all its characteristics. Complex systems are characterized by several distinct properties that arise from the interactions among their components. These properties make complex systems difficult to model and predict, as they often exhibit behaviors that are not apparent when examining individual parts in isolation.

We enumerate their properties in accordance with the principles outlined by Arshinov and Fuchs [5]. Emergence and self-organization are the two main concepts when we consider a complex system. Aspects of emergence are: synergism, novelty, irreducibility, unpredictability, coherence/correlation, and historicity. Self-organization includes the following aspects: systemness, complexity, cohesion, openness, bottom-up-emergence, downward causation, nonlinearity, feedback loops, circular causality, information, relative chance, and hierarchy.

Complex systems can be rigorously examined using a wide range of methodologies and techniques tailored to capture their core properties above mentioned. Here are some fundamental approaches for studying these multifaceted systems: (1) data analysis and modeling tools (such as time series analysis, agent-based models, cellular automata, and information theory); (2) network and systems analysis (including network theory, nonlinear dynamics, self-organization, and emergence); (3) interdisciplinary approaches (such as statistical mechanics and physics, game theory, and adaptive systems); and (4) computational and simulation methods (including differential equations, Markov processes, and graph theory) [6].

PE is deeply connected with foundational ideas in computation and information theory, tracing back to Alan Turing’s pioneering work on algorithmic complexity and pattern recognition. His conceptualization of computation and his exploration of information processing laid the groundwork for modern measures of complexity and entropy, including PE, which can be seen as a practical tool to quantify the unpredictability and information content in sequences generated by computational or natural processes. Thus, PE not only serves as a robust measure of complexity in nonlinear time series but also embodies the legacy of Turing’s contributions to understanding complexity and information in dynamic systems. Tere are some points where is seen connection. Algorithmic complexity and Turing machines. Turing’s universal machine underpins algorithmic (Kolmogorov) complexity, which measures the shortest program length needed to reproduce a sequence on a universal Turing machine. While Shannon entropy quantifies the average uncertainty of a random variable, algorithmic complexity captures the specific information content of individual sequences. Indirect influence on entropy applications. Turing’s statistical insights, such as coverage deficits (used to estimate unseen species probabilities), were later adapted to estimate Shannon entropy in ecological contexts. Comparative analysis of measures. As it is said, the Shannon entropy is a probabilistic measure of average uncertainty, while algorithmic complexity is a deterministic measure of individual sequence complexity. Hybrid approaches like the Block Decomposition Method (BDM) bridge these concepts by combining local algorithmic complexity estimates with Shannon entropy for larger-scale patterns’. In conclusion, while Shannon’s entropy quantifies uncertainty in information theory, Turing’s insights into randomness and unpredictability provide a foundational framework for understanding noise tolerance in communication systems [7,8,9,10,11].

### 1.2. Permutation Entropy and Time Series Analysis

Natural fluids are exemplary physical complex systems. Hydrologists design their models heuristically, varying in levels of sophistication. These models enable understanding of complex hydrological processes, enhance predictive capabilities regarding hydrological phenomena, identify significant trends, facilitate flood risk assessments, improve access to data quality, and inform effective water resource management strategies. The complexity of a complex system cannot be fully modeled. It is essential to know that even models have their complexities, which can raise questions about the results’ reliability. Across all scientific disciplines, the available information is generally limited to what has been observed or measured, typically presented as (i) equidistantly ordered numbers or (ii) converted into a time series arrangement. Time series are a natural source of information about phenomena, yet they convey a message that is locked. From these time series, it is possible to calculate complexity measures for both low-dimensional systems, such as coded strings of symbols (e.g., measured quantities, text, computer code, proteins), and high-dimensional systems (e.g., ecosystems, brain activity, telecommunications networks, computer systems, biological systems, social structures), which require methods from network science [12]. Recent efforts have aimed to establish a complexity hierarchy between the primary variable (master variable) and other system components that affect it [13].

Historically, Kolmogorov complexity (approximated via the Lempel-Ziv algorithm [14]) and Shannon entropy were the primary measures used to assess complexity until the early 21st century. The introduction of permutation entropy (PE) by Bandt and Pompe [15] in 2002 expanded the toolkit for complexity analysis. It is an intrinsic measure of complexity in time series analysis, characterized by its unique properties and advantages over traditional methods. Its key features include a foundation in ordinal analysis, robustness against noise, invariance properties, and computational efficiency. This information measure has quickly found applications across a wide range of scientific fields: biomedical sciences [16,17,18,19], econophysics [20,21], signal processing [22,23], climate research [24,25], laser physics and geophysics [22,26], machine learning [23] as well as in hydrology ([27,28,29,30,31], among many others).

In this paper, Section 2 provides a brief overview of the development of PE from 2002 to 2024, while Section 3 presents an outlook on the application PE in hydrology.

## 2. Permutation Entropy: Its Advantages and Limitations

PE [15] is a method for analyzing the complexity of dynamical systems based on the relative ordering of values in a time series. It has proven effective in distinguishing regular, stochastic, and chaotic behaviors due to its robustness against observational noise and simplicity of implementation. The method relies on ordinal patterns—permutations representing the relative order of data points—and calculates entropy based on their probability distribution [32]. Despite its favorable characteristics, PE remains not fully understood on a theoretical level. This gap in theoretical comprehension underscores the need for further research to fully elucidate its properties and potential applications [33]. There exist time series for which the use of PE is not sufficient to capture both randomness and structure in time series data. Rosso and co-workers [34,35] developed an advanced statistical complexity measure designed to analyze the dynamics of systems and time series. It builds upon the earlier LMC (López-Ruiz, Mancini, and Calbet) statistical complexity by refining the distance component in the probability space. This measure, known as MPR (Martín, Plastino, and Osvaldo Rosso) complexity combines normalized Shannon entropy (quantifying disorder) with a disequilibrium term (capturing deviation from uniformity) to assess both randomness and structural correlations in a system’s probability distribution. The MPR complexity has applications in many scientific disciplines time series as well as in hydrology in analyzing river flow dynamics [34,35,36,37].

PE of a time series ${\left\{\left\{x_{t}\right\}\right\}}_{i=1}^{N}$ is calculated through the following steps.

1. Time series embedding. Given a time series ${\left\{\left\{x_{t}\right\}\right\}}_{i=1}^{N}$ define embedding dimension n (also called order or pattern length) and delay τ (step between consecutive points). Create vectors:

$$v_{t}=(x_{t},x_{t+\tau },x_{t+2\tau },\ldots ,x_{t+(n-1)\tau }).$$

For example, with n=3 and τ=1, the vector for t=1 is $\left(x_{1},x_{2},x_{3}\right)$.

2. Ordinal pattern extraction. For each vector $v_{t}$, determine the permutation π $=(r_{0},r_{1},r_{2},\ldots ,r_{n-1})$ representing the ranks of its elements in ascending order. Example: For $v_{t}=(9,6,11)$, ranks are (2,1,3), corresponding to permutation π =(2,1,3).

3. Probability distribution calculation. Compute the relative frequency of each permutation pattern $\pi_{j}:$

$$p_{j}=\frac{\mathrm{Number}\;\mathrm{of}\;\mathrm{occurrences}\;\mathrm{of}\;\pi_{j}}{N-(n-1)\tau }.$$

For n=2, possible permutations are (0,1) and (1,0). If (0,1) occurs 4 times in 6 trials $p_{\left(0,1\right)}=$2/3.

4. Shannon entropy computation. Calculate the Shannon entropy of the permutation distribution:

$$H_{n}=\sum_{j=1}^{n!}p_{j}{log}_{2}p_{j}$$

For the n=2 example above:

$$H(2)=-\left(\frac{2}{3}{log}_{2}\frac{2}{3}+\frac{1}{3}{log}_{2}\frac{1}{3}\right)\approx 0.918\;\mathrm{bits}.$$

5. Normalization. Normalize by the maximum entropy ${log}_{2}(n!)$:

$$h_{n}=\frac{H(n)}{{log}_{2}(n!)}$$

This ensures $0\leq h_{n}\leq 1$, where 1 indicates maximum randomness.

Advantages and Limitations. This information measure provides substantial theoretical and practical advantages, establishing itself as a critical methodology in comprehensive time series analysis. Here are some key advantages. (1) Intuitive background. PE is considered an intuitive measure because it captures the complexity and randomness of time-series data in a straightforward and accessible manner. It is based on the idea of analyzing the order patterns within a sequence of data points rather than their exact numerical values. This approach makes it easier to understand and apply, as it directly reflects the likelihood of observing specific arrangements or patterns in the data [38]. (2) Computational efficiency is observed through several key factors: high-speed calculations, low computational overhead and minimal memory requirements. These features make PE efficient for analyzing high-frequency data and provide reliable entropy estimations from relatively small sample sizes [16,38]. On the other hand, calculating PE becomes computationally challenging at higher embedding dimensions (order) due to the exponential growth in the number of possible ordinal patterns. For an embedding dimension m, there is m factorial of possible patterns, which significantly increases memory and computational requirements as m grows [39,40,41]. As a result, we encounter two limitations: memory bottlenecks and computational complexity. Firstly, storing and processing the large number of ordinal patterns requires substantial memory. This limitation can hinder practical computation on systems with limited resources [39,40]. Secondly, in the case of computational complexity, related to the amount of resources needed for running an algorithm, there is a need to calculate probabilities for all factorial of m patterns. This increases computation time exponentially, making it impractical for very high dimensions [39,40]. To address these challenges, researchers have proposed modifications such as network-based PE or non-uniform embedding techniques to optimize calculations and reduce computational demands [41]. (3) Robustness to noise. PE demonstrates remarkable robustness against noise, highlighting its ability to deliver consistent and reliable performance even in the presence of noise within input data or the surrounding environment. This critical property plays an essential role across a wide range of fields, including auditory perception, machine learning, and signal processing, as well as in specialized domains such as biomedical engineering, financial analytics, and analysis of chaotic signals affected by a weak observational noise [16,21,42,43]. (4) Minimal parameter requirements. In contrast to many other techniques, PE significantly reduces the complexity typically associated with extensive parameter tuning, allowing users to apply it more readily across various contexts [42,44,45,46,47]. (5) Discrimination of dynamics. One of the most remarkable properties of PE is its ability to distinguish between different types of dynamical behavior, such as periodic and chaotic dynamics [15,38]. This capability stems from its distinctive approach to quantifying the order relations among data points in a time series. Numerous studies have further explored and enhanced this property, highlighting its versatility and effectiveness in analyzing complex systems [48,49,50,51,52].

Despite its advantages, PE does have some drawbacks, which we will briefly outline. (i) Loss of amplitude information. A notable limitation of PE is its inability to incorporate amplitude information from the time series. While PE effectively captures the ordinal patterns, it disregards the actual magnitudes of the data points. This omission can result in the loss of critical information, particularly in applications such as signal time series analysis, where amplitude variations often carry significant meaning [24]. Various modifications to the standard PE have been proposed to incorporate amplitude information to address this issue. For example, improved PE versions integrate ordinal relations and amplitude data, enabling a more comprehensive analysis [48,53]. These enhancements allow for retaining crucial amplitude-related characteristics while preserving the ability to capture the underlying order dynamics [54,55]. (ii) Handling equal values. The presence of equal values in a time series can significantly distort the ordinal patterns upon which PE depends. This distortion may result in inaccuracies in entropy calculations. To moderate the issues caused by equal values, several strategies have been proposed [56,57]. (iii) Heuristic methods in parameter selection. The effectiveness of PE is highly dependent on the careful selection of key parameters, such as the permutation dimension and embedding delay. These parameters play a critical role in optimizing the performance of PE. However, their selection often relies on heuristic methods—practical, trial-and-error approaches that prioritize feasible solutions over theoretically optimal ones. While these methods can be effective in practice, they may lack consistency and precision, underscoring the need for more systematic and data-driven parameter-tuning strategies [42,46,47]. This is an appropriate point to reference the paper of Riedl et al. [42], which offers the most valuable guidance on addressing this issue. They explored various fields where PE has been applied and provided a guide on how to choose appropriate parameters for different PE applications. According to them the embedding dimension n and time delay τ must be chosen carefully depending on the nature of the data. For time series with inherent cycles or event-based processes, a delay τ=1 is commonly appropriate, while for continuous dynamical systems, higher delays can reveal more structure, and the optimal τ can be identified by minimizing PE. The embedding dimension n should be as high as possible but constrained by the length of the time series, following the recommendation N>5N!  to ensure reliable statistics. The multiscale complexity-entropy causality plane represents an advanced and effective approach for selecting permutation entropy parameters, enabling the characterization of time series complexity across multiple temporal scales and enhancing the discrimination between chaotic and stochastic dynamics [58]. (iv) Inability to differentiate between distinct patterns within the same motif. Some waveforms, such as electroencephalograms (EEG) [59], can be represented by a series of ordinal patterns known as motifs, which are derived from the ranking of values in subsequence time series. PE is designed to capture the relative occurrence of these motifs; however, it struggles to differentiate between distinct patterns within a given motif. This limitation can be addressed through the use of Weighted-Permutation Entropy (WPE), a modified version of PE proposed by Fadlallah et al. [60]. (v) Sensitivity to certain processes. Recent studies have indicated that traditional PE may struggle to capture slow-changing signals’ dynamics accurately. This limitation is particularly significant when analyzing data from systems that evolve gradually over time, as it can lead to an underestimation of their complexity [24,57,61,62]. (vi) Complexity in interpretation. PE is a powerful tool for measuring complexity, but its interpretation can be challenging, particularly in diverse applications such as EEG analysis or financial time series. The complexity of these systems often requires a nuanced understanding of the underlying dynamics, and PE values alone may not provide sufficient insight. Therefore, clear and meaningful conclusions cannot be drawn without additional context or complementary analyses [63].

## 3. Permutation Entropy in Hydrology: Diverse Applications and Insights

PE has significantly expanded its application across various fields of hydrology, primarily due to its effectiveness in analyzing the complexity and dynamics of hydrological time series data. Since 2002, the number of research papers utilizing PE in hydrology has increased steeply. This remarkable growth can be attributed to several key factors: advancements in the standard methodology of PE, the development of models that effectively incorporate this metric and significant progress in computational techniques, including neural networks and artificial intelligence (AI). Selecting a single effective approach to review the applications of PE in hydrology was challenging. This process was carried out in the following steps. Categorization: We categorized the uses of PE across various subfields, examining its role in runoff prediction, streamflow analysis, water level forecasting, assessment of hydrological changes, and the impact of infrastructure on hydrology. Inclusion criteria: We included papers primarily or partially focused on PE. Runoff predictions. He et al. [64] proposed a non-stationary monthly runoff prediction model for optimizing agricultural water management. This model is particularly useful for large irrigation areas along river basins, addressing the spatial and temporal variations in runoff. PE was employed to assess the complexity and randomness of time series in the context of monthly runoff prediction in an oasis irrigation area. It helped analyze the chaotic characteristics of runoff sequences, which are influenced by non-stationary and stochastic factors. PE facilitated the decomposition of runoff sequences into components with varying complexity, aiding in more precise modeling. In the paper is noted that while PE is effective for identifying randomness and complexity, it may not fully capture long-term dependencies in time series data. The computational cost can increase when dealing with high-dimensional or very longtime series. A single model cannot fully address the challenges posed by the non-stationarity of runoff. In the hybrid model developed by Wang et al. [65], PE was integrated with Variational Transform Finite Element Modal Decomposition (TVFEMD) and Temporal Convolutional Networks (TCN) to form the TVFEMD-PE-TCN model. This model classifies intrinsic mode functions (IMFs) derived from runoff sequences into high- and low-frequency components based on their complexity.

It quantified randomness and helped isolate spurious elements, improving the accuracy of predictions by distinguishing between chaotic and structured components. A limitation of this model is the effectiveness of PE that can be influenced by noise or large fluctuations in data, which might lead to challenges in accurately clustering low-frequency components. Excessive decomposition using TVFEMD combined with PE can increase computational complexity and may introduce errors. By decomposing runoff data into multiple scales; the model facilitates a more detailed analysis and improves forecasting capabilities [66]. In this study, PE was used within a multi-scale two-phase hybrid model for runoff prediction to measure the complexity of decomposed components. It helped identify chaotic patterns and contributed to improved model performance by enabling better separation of noise from meaningful signals. Similar to other studies, the reliance on PE for complexity assessment might not fully address issues related to long-term trends or dependencies in runoff data. The method’s sensitivity to parameter selection (e.g., embedding dimension, delay time) could lead to inconsistencies if not carefully optimized.

The impact of climate change on streamflow complexity is a critical issue for hydrological modeling, runoff predictions, and water resource management. In Shen et al. (2020) [67], complexity is operationalised through the lens of PE, a nonlinear time-series analysis tool that quantifies the degree of disorder in a system [68], that with higher entropy values indicating greater unpredictability. For streamflow, this translates to a system dominated by stochastic or chaotic processes, whereas lower entropy reflects periodic or stable behavior.

The findings in Shen et al. [67] indicate a substantial increase in streamflow complexity since 1972, which is attributed to climate change. The study observed a marked rise in PE values post-1972, signaling heightened unpredictability in streamflow dynamics. This complexity shift correlates with warming temperatures and increased precipitation, leading to altered hydrological processes such as glacier melt and permafrost thaw. The increased complexity has significant implications for runoff predictions, as the hydrological system becomes more non-linear and less predictable. This poses challenges for water resource management and forecasting in the region, especially under accelerated climate change scenarios.

Streamflow analysis. The analysis of streamflow time series on different time scales using PE has been widely recognized as an efficient method since its introduction. From the beginning, PE was mainly applied to distinguish between chaotic and stochastic behaviors in hydrological systems. Over time, as the methodology advanced, PE became instrumental in understanding the complexity of streamflow dynamics. This progress has been particularly significant in exploring the impacts of climate change and human activities on hydrological phenomena.

The pioneering work in applying PE to analyze climate change impact in hydrology is a paper by Hao et al. (2007) [25]. The authors evaluated: (i) climate complexity (higher PE indicates greater unpredictability in climate patterns); (ii) regional variations (variations of PE across regions suggest how environmental factors affect climate complexity); and (iii) temporal trends (noted temporal variations of PE point out changes in climate complexity) in Yunnan Province for the period 1971–2000. However, the limitations of PE in terms of parameter sensitivity and mechanistic interpretation underscore critical areas that require refinement in future applications. Specifically, optimizing embedding parameters and integrating PE with other metrics to provide expanded awareness into underlying physical processes will be essential for enhancing its utility in complex systems analysis.

Mihailović et al. (2014) [69] analyzed monthly streamflows of two mountain rivers in Bosnia and Herzegovina. PE was utilized in conjunction with other metrics, including Kolmogorov complexity and sample entropy, to assess the randomness and complexity of monthly streamflow time series. By examining ordinal patterns within the data, PE provided valuable comprehensions into the nonlinear dynamics and turbulence of environmental fluid flows and loss of complexity of mountain rivers. The authors computed PE values for three distinct sub-periods—1926–1945, 1946–1965, and 1966–1990—to investigate temporal changes in complexity. Notably, decrease in complexity during 1946–1965 was linked to increased human interventions (such as water consumption) and climate change impacts. To evaluate the robustness and reliability of PE across different data configurations, its values were analyzed as a function of embedding dimension and time series length. The study revealed a reduction in complexity during the mid-20th century, coinciding with heightened human activities and climate-related changes. Importantly, PE values aligned with trends observed in other metrics, such as Kolmogorov complexities, underscoring its reliability in detecting changes in complexity. Additionally, while the statement that “the loss of complexity in river streamflow is particularly characteristic of mountain rivers” holds true within contexts where mountain rivers are subject to anthropogenic or climatic pressures, it is essential to recognize that complexity loss is not an intrinsic feature of mountain rivers. Rather, it is a consequence of external disturbances that alter their natural hydrogeomorphic processes.

The paper [70] studied the influence of anthropically induced effects on streamflow dynamics. PE is utilized alongside statistical complexity to quantify the randomness and complexity of streamflow time series data. By analyzing ordinal patterns within the data, PE detects changes in dynamical behavior induced by human interventions, such as dam construction or water management practices. PE values are computed for streamflow time series from multiple hydrological stations to evaluate the degree of randomness and chaos across different regions. Additionally, the complexity-entropy causality plane (CECP) is employed to visualize the relationship between PE and statistical complexity, offering a robust framework for distinguishing chaotic and stochastic dynamics. Through comparative analysis of PE values across stations and time periods, the study identified anthropically induced effects on streamflow dynamics. These effects include reduced variability or increased regularity resulting from controlled water flow due to human activities.The limitation of this paper lies within the framework of the drawbacks associated with PE.

Suriano et al. [71] explored the impact of building the Yacyretá Dam on the daily streamflow of the Parana River (Argentina), using information quantifiers such as statistical complexity and PE. The authors analyzed daily streamflow time series recorded during the 1929–2010 period, encompassing the 1973–1979 period when the dam was built. The main objective was to identify key details of the dynamics of the analyzed time series to differentiate the degrees of randomness and chaos. The PE is used with the probability distribution of ordinal patterns and the Jensen-Shannon divergence to calculate the disequilibrium and the statistical complexity. Daily streamflow series at different river stations were analyzed to classify the different hydrological systems. The CECP and the representation of the Shannon entropy and Fisher information measure (FIM) show that the daily discharge series could be approximately represented with Gaussian noise, but the variances highlight the difficulty of modeling a series of natural phenomena. It was found that the original and deseasonalized streamflow time series had different complexities and entropy patterns before the building of the dam.

Minimizing the cost monitoring of daily streamflow data is an important issue in hydrology [72]. To address this issue, the authors applied joint PE to data recorded during the period 1989–2016 at twelve gauging stations on the Brazos River, Texas, USA. They found that the best cost efficiency is achieved by performing weekly measurements, in comparison with daily ones, which exhibit information redundancy.

As highlighted above, the MPR complexity metric has demonstrated broad applicability across diverse scientific domains. Within hydrological research, it has emerged as a particularly valuable tool for characterizing nonlinear dynamics in river flow systems, enabling deeper insights into flow regime transitions, multiscale interactions, and climate-driven behavioral shifts [34,35,36,37].

Hydrological changes assessment. To clarify the role of PE in hydrological changes assessment, note that it involves evaluating alterations in the water systems of landscapes, often due to factors like climate change, land use intensification, and water management practices. This assessment is crucial for understanding how these changes impact ecosystems and for developing strategies to mitigate adverse effects. PE is typically not directly applied for hydrological changes assessment, but it is utilized to analyze the complexity and randomness of various environmental and hydrological time series data, thereby providing valuable insights into the assessment of hydrological changes [73]. We will briefly discuss some of these papers. In [71] the authors applied PE to analyze daily streamflow dynamics in Argentine rivers, examining randomness and complexity in hydrological systems. It also evaluates the impact of human interventions like dam construction. Although study in [74] is focused on paleoclimate records, it employs PE to identify anomalies in water-isotope data from ice cores, a method that could potentially be applied to hydrological time series data. Zhang et al. [75] investigated temperature fluctuation complexity across China using PE, analyzing spatial and temporal variations. Their work provides a methodological framework that can be adapted for hydrological assessments, particularly in studying climate-driven changes. In [76], the authors discussed methodologies for evaluating hydrological responses to climate change, highlighting the potential applications of entropy-based measures, such as PE. The significance of this study lies in its provision of insights into how climate change affects hydrological systems, an area where PE could be effectively applied to analyze complexity and randomness in hydrological time series data.

Water level forecasting. Accurate water level forecasting is essential for effective flood control, agricultural planning, and infrastructure management. Recent research has demonstrated that integrating positional encoding with advanced machine learning models significantly enhances prediction accuracy in hydrological systems. These state-of-the-art models consistently outperform traditional forecasting methods, providing more reliable predictions of water level fluctuations. Thus, the Gated Recurrent Unit—Temporal Convolutional Network—Transformer (GRU–TCN–Transformer) AI-based model [77] combines singular spectrum analysis and complete ensemble empirical mode decomposition with adaptive noise to decompose water level data into frequency components. It uses PE to classify these components into high- and low-frequency sequences, predicting the high-frequency sequences with a GRU model and the low-frequency sequences with a TCN–Transformer hybrid.

The paper by Sun et al. [78] utilizes PE as a key analytical tool to enhance the interpretability of echo state networks in time series prediction tasks. By leveraging PE, the authors develop a novel framework to measure reservoir dynamics and projection capacity. They employ PE alongside multiscale complexity–entropy analysis to quantify reservoir richness and neuronal dynamics, making it a central component of their methodology. Although this paper does not specifically focus on water level forecasting, the techniques and insights it provides could be adapted for such applications if combined with suitable predictive models. For instance, PE could be used to analyze the complexity of water level time-series data, which could then inform model selection or preprocessing steps in forecasting frameworks. This approach could potentially improve the accuracy and reliability of water level predictions by better understanding the underlying dynamics of the data.

In closing, we expanded the discussion by presenting a concise bibliographic analysis aimed at: (1) offering practical recommendations for the application of PE in hydrology, (2) highlighting current research gaps, and (3) outlining future directions to advance this field.

Some recommendations for applying PE in hydrology are as follows: (i) Standardize data preprocessing by implementing consistent procedures for noise reduction, normalization, and handling of missing data to enhance the reliability of PE in hydrological time series analysis [79]. (ii) Optimize parameter selection by customizing the embedding dimension and time delay parameters to fit the specific hydrological context, taking into account the system’s temporal variability and data resolution. This approach ensures that the reconstructed phase space accurately captures the underlying dynamics relevant to hydrological processes and the scale of observations [42]. (iii) Integrate PE with network design by using PE alongside entropy-based criteria to select monitoring stations that maximize information gain while minimizing redundancy, thereby enhancing network efficiency [72]. (iv) Apply multi-scale analysis by employing multi-scale PE to capture complex hydrological processes occurring at different temporal scales, such as seasonal or event-driven dynamics. (v) Case studies: Conduct empirical studies on diverse hydrological systems (e.g., groundwater, surface water) to demonstrate PE’s practical utility and guide its application [80].

In our opinion, the current research on applying PE in hydrology exhibits the following research gaps: (i) Limited spatial-temporal scaling studies: Further research is needed to understand how PE results change with varying spatial and temporal resolutions of hydrological data. This understanding is crucial for effective network design and data interpretation. (ii) Underexplored hydrological domains: The application of PE remains limited in areas such as groundwater monitoring, snowmelt processes, and coupled human-natural water systems. (iii) Interpretability and practical guidelines: Frameworks that translate PE metrics into actionable hydrological insights or decision-making tools for practitioners are lacking. (iv) Tool development and accessibility: The scarcity of user-friendly software tools for hydrologists hinders the broader adoption of PE.

The next steps to advance the field of hydrology can be outlined as follows: (1) Develop hybrid methodologies: Combine PE with machine learning techniques (e.g., Long Short-Term Memory (LSTM) networks) or causal inference methods to enhance predictive power. (2) Establish benchmark datasets: Curate labeled hydrological datasets with known dynamical regimes to test PE’s robustness across different climates and basins. (3) Foster interdisciplinary collaborations: (4) Bridge nonlinear dynamics experts and hydrologists through workshops or shared challenges. Otherwise, the gap in their understanding will only grow larger. (5) Create best-practice guidelines: Publish a community-driven white paper on PE applications in hydrology, addressing common pitfalls such as overfitting and miss parameterization.

## 4. Conclusions

In this paper we reviewed the use of PE in hydrology. The main conclusions are as follows:

(1) Hydrologists typically analyze complexity by applying information measures to measured time series—a natural source of information—instead of using heuristic hydrological models. PE is perhaps the most widely used complexity measure in hydrology.
(2) We briefly discuss its advantages, which include an intuitive background, computational efficiency, robustness to noise, and minimal parameter requirements, and discrimination of dynamics. However, PE also has several drawbacks: loss of amplitude information, difficulties in handling equal values, reliance on heuristic methods for parameter selection, inability to differentiate between distinct patterns, sensitivity to certain processes, and complexity in interpretation.
(3) We reviewed the diverse applications of PE in hydrology, categorizing its uses across various subfields. Specifically, we examined PE’s role in runoff prediction, streamflow analysis, water level forecasting, assessment of hydrological changes, and evaluating the impact of infrastructure on hydrology.
(4) Finally, we (1) offer practical recommendations for applying PE in hydrology, (2) highlight current research gaps, and (3) outline future directions to advance this field.
